# A home-based lifestyle intervention program reduces the tumorigenic potential of triple-negative breast cancer cells

**DOI:** 10.1038/s41598-024-52065-9

**Published:** 2024-01-29

**Authors:** Giulia Baldelli, Valentina Natalucci, Carlo Ferri Marini, Davide Sisti, Giosuè Annibalini, Roberta Saltarelli, Matteo Bocconcelli, Veronica Gentilini, Rita Emili, Marco Bruno Luigi Rocchi, Francesco Lucertini, Elena Barbieri, Giorgio Brandi, Mauro De Santi

**Affiliations:** 1 Unit of Pharmacology and Hygiene, Department of Biomolecular Sciences, University of Urbino Carlo Bo, 61029 Urbino, Italy; 2 Division of Exercise and Health Sciences, Department of Biomolecular Sciences, University of Urbino Carlo Bo, 61029 Urbino, Italy; 3 Medical Oncology, Hospital Santa Maria della Misericordia di Urbino, 61029 Urbino, Italy

**Keywords:** Health care, Breast cancer, Cancer models, Cancer prevention

## Abstract

Translational research for the evaluation of physical activity habits and lifestyle modifications based on nutrition and exercise has recently gained attention. In this study, we evaluated the effects of serum samples obtained before and after a 12-week home-based lifestyle intervention based on nutrition and exercise in breast cancer survivors in terms of modulation of the tumorigenic potential of breast cancer cells. The home-based lifestyle intervention proposed in this work consisted of educational counselling on exercise and nutritional behaviors and in 12 weeks of structured home-based exercise. Triple-negative breast cancer cell line MDA-MB-231 was cultured in semi-solid medium (3D culture) with sera collected before (PRE) and after (POST) the lifestyle intervention program. Spheroid formation was evaluated by counting cell colonies after 3 weeks of incubation. Results show a slight but significant reduction of spheroid formation induced by serum collected POST in comparison to those obtained PRE. Moreover, statistical analyses aimed to find physiologic and metabolic parameters associated with 3D cell proliferation revealed the proliferative inducer IGF-1 as the only predictor of cell tumorigenic potential. These results highlight the importance of lifestyle changes for cancer progression control in a tertiary prevention context. Translational research could offer a useful tool to identify metabolic and physiological changes induced by exercise and nutritional behaviors associated with cancer progression and recurrence risk.

## Introduction

Epidemiological evidence shows that individuals who maintain an active lifestyle and recommended levels of physical activity (PA) have a remarkably lower risk of non-communicable diseases, including cancer^1^. In a tertiary prevention approach, there is growing evidence that lifestyle factors such as poor diet, obesity, and sedentary behavior play an important role in determining rates of recurrence and prognosis among breast cancer (BC) survivors^2^.

Cohort studies conducted on patients in post-treatment follow-up have shown that exercise may be particularly suitable in this phase, as it improves psychophysical and cardiometabolic health^3,4^. Nutritional aspects are also important in cancer post-treatment follow-up due to the modulation of hormonal levels related to cancer progression, such as the insulin-like growth factor-1 (IGF-1) and those induced by hyperglycemia and abdominal fat^5,6^. It has been shown that the risk of recurrences could be prevented through proper nutrition, weight control, and recommended levels of PA^7^.

In this context, the translational approach based on in vitro assays has recently attracted growing attention, as described by recent reviews and meta-analyses^8–10^. These studies show that acute bouts of exercise lead to serological changes that impact the proliferation capacity of cancer cells in vitro, as well as cell viability and tumorigenic potential. Several in vitro models have been used in different studies and collectively support the notion that PA plays an important role in modulating BC progression. However, authors strongly support the need for 3D cell models, which are more physiologically relevant, allowing researchers to expose BC cells to different types and levels of physical activity in vitro to understand the mechanistic pathways involved, too^9,10^. Translational research applied to exercise offers a great opportunity to analyze the effects of exercise on cancer cell behavior, providing precise information about the time course of the responses.

Cancer recurrences occur if cancer is found after treatment and/or surgery and after a period of time when it cannot be detected. The cellular mechanism of this phenomenon is based on metastatic dormancy principles, described as the period after treatment when residual malignant cells become detectable either as a recurrent local or metastatic disease^11^. To mimic the biological mechanisms of cancer dormancy in vitro (e.g., single-cell dormancy and re-growth), we have developed a 3D cellular model that involves cell growth in the semisolid medium^12–14^. Using this method, we have previously shown that exercise-conditioned sera from young, healthy women significantly reduced the ability of triple-negative BC (TNBC) cells to form spheroids in soft agar in comparison to sera taken at rest^12,13^.

In this study, we evaluated the effects of serum samples collected from 30 BC survivors before (PRE) and after (POST), a 12-week home-based lifestyle intervention (LI) program on triple-negative BC cell proliferation in the 3D culture model. Moreover, the associations between spheroid formation and anthropometric, body composition, PA level (PAL), and metabolic parameters were analyzed.

## Materials and methods

### Population

Participants in this study were 30 BC survivors women enrolled in the first recruitment of the MoviS trial (protocol: NCT04818359)^15,16^. The study followed the guidelines of the Helsinki Declaration for research with human volunteers (1975) and was approved by the Human Research Ethics Committee of the University of Urbino Carlo Bo (Protocol N 21 of 10 July 2019). All participants’ written signatures of the informed consent were obtained prior to the start of the study. Recruitment details are described in Natalucci et al.^3^. Briefly, inclusion criteria were: ≤ 12 months post-surgery and post chemo- or radio-therapy adjuvant; stage 0 to III BC without metastases or recurrences diagnosis at recruitment; aged 30–70 years; non-physically active (i.e., not engaged in regular activity according to the International Physical Activity Questionnaire Short Form (IPAQ-SF)^17,18^ for at least 6 months; with a high risk of recurrence^19,20^. Exclusion criteria were: disabling pneumatological, cardiological, neurological, orthopedic comorbidities, or mental illnesses that prevent exercise performance; treatment with beta-blockers, non-dihydropyridine calcium channel blockers, or amiodarone due to their potential effect on heart rate response to exercise; treatment with antidepressant drugs.

### Study design

This study aimed to evaluate the effect of a 12-week LI program on TNBC cell tumorigenic potential in a 3D culture in vitro model. Serum samples were collected from the first enrollment of the MoviS trial (NCT04818359) which planned to investigate a 12-week LI on Quality-of-Life changes, dividing participants in two arms (i.e., intervention [IA] and control [CA] arm); however, due to the imposed COVID-19 pandemic restrictions, the exercise training for the IA had to be adapted to a home-based exercise program (Human Research Ethics Committee of the University of Urbino Carlo Bo; Protocol N. 21, 10 July 2019; Protocol amendment N. 29, 22 April 2020).

In detail, both arms received a 12-week LI based on nutritional and exercise educational counselling (phase I), and the IA only followed MoviS training for 12 weeks (phase II). Educational counselling during phase I was organized in structured meetings lasting about 1 h (at baseline) in which participants of both arms received exercise and nutrition counselling by an exercise specialist, oncology nutritionist, and psycho-oncologist, respectively. Exercise advice was based on current guidelines^21^ with practical suggestions to apply to daily life, while the nutritional advice was based on the Mediterranean diet^22^. Particularly, it was suggested to consume meals mainly containing whole grains, legumes, vegetables, and seasonal fruit; fish was indicated as the preferred animal protein source. The consumption of preserved meats, sugary drinks, and ultra-processed foods was discouraged. In addition, women received a registration on the DianaWeb platform^23,24^. IA participants received the MoviS training, in which for the first 3 weeks, sessions were monitored according to the original protocol (2 days/week of directly supervised exercise and 1 day/week of remotely monitored exercise). However, from the 4th week of the MoviS training, COVID-19 pandemic restrictions imposed changes in the protocol, and the study kept on with only a remotely supervised exercise program. From weeks 4 to 12, IA participants received weekly phone calls from the exercise specialist with the aim of maintaining the 3 sessions per week of aerobic exercise and received a prescription and personalized feedback according to the training logs. For each home-based exercise session, IA participants could choose to exercise indoors using treadmills or stationary bikes and outdoors without any equipment. Regardless of the exercise modality, the sessions were performed at individualized exercise intensity (e.g., walking speed and grade or cycling wattage), allowing each participant to reach and maintain the prescribed target heart rate during the training sessions. Furthermore, all the participants (IA and CA) received weekly phone contacts to remind healthy nutritional and exercise lifestyles. The flow diagram of the study design is reported in Supplementary Fig. S1.

### Physiological and metabolic parameters assessments

Physiological and metabolic parameters before (PRE) and after (POST) the home-based LI were collected as described in Natalucci et al.^3^. Briefly, the body composition was assessed through BMI measurements, and participants’ predicted maximal heart rate and ${\dot{\text{V}}\text{O}}_{2\max }$ were estimated by a personalized submaximal incremental walking test on a treadmill^25–28^. Moreover, blood glucose, insulin, triglycerides, HDL, LDL, and total cholesterol concentrations were determined by colorimetric assays on Beckman Coulter AU Analyzers, and the homeostasis model assessment was used to estimate insulin resistance (HOMA-IR)^3^. Serum concentrations of IGF-1 and IGF-1 binding protein 3 (IGFBP3) were measured with a solid-phase, enzyme-labeled chemiluminescent immunometric assay with the IMMULITE 2000 analyzer (Siemens Healthcare s.r.l., Italy), according to the manufacturer’s protocols. The IGF-1/IGFBP3 molar ratio was calculated according to the formula: [IGF-1 (ng/mL) × 0.13]/[IGFBP3 (ng/mL) × 0.035]^29^. Serum high sensitivity C-Reactive Protein (hs-CRP) levels were measured by the Beckman Coulter AU System CRP Latex reagent on Beckman Coulter AU Analyzers.

Adherence to a Mediterranean diet was assessed by the MeDiet modified questionnaire, which was analyzed using the MeDiet Score^30^. PAL was assessed by using the interviewer-administered IPAQ-SF questionnaire^17,18^. Quantitative variables were reported as mean ± standard deviation; parameters measured were BMI, PAL, *V*O_2max_, glucose, insulin, HOMA-IR, IGF-1, IGFBP3, triglycerides, total cholesterol, HDL, LDL, troponin, creatine kinase, and hs-CRP.

### Cell culture

The TNBC MDA-MB-231 cell line was obtained from the American Type Culture Collection (ATCC, Rockville, MD, USA). Cells were maintained in culture in Dulbecco’s Modified Eagle Medium (DMEM) with high glucose, containing 4500 mg/L of glucose and supplemented with 10% v/v fetal bovine serum (FBS), 1× MEM non-essential amino acid solution, 2 mmol/L l-glutamine, 1 mM Na-Pyruvate, 0.1 U/L penicillin, and 0.1 mg/mL streptomycin. Cells were maintained in a humidified incubator at 37 °C and at 5% CO_2_, during a maximum of 15 passages. During the experiments, the culture medium was replaced by DMEM without red phenol and without glucose, then supplemented with a physiological concentration of glucose (80 mg/dL; 4.4 mmol/L). Moreover, 10% v/v FBS was replaced with 5% v/v human serum (HS) obtained before (PRE) or after (POST) the LI program. All cell culture materials were purchased from Corning Inc. (New York, USA).

### Three‑dimensional (3D) cell culture assay (soft agar)

The effect of HS conditioned by the home-based LI program on the tumorigenic potential of TNBC cells was evaluated in terms of 3D spheroid formation in anchorage-independent culture conditions. The MDA-MB-231 cells were cultured in soft agar with 5% v/v HS obtained before (PRE) or after (POST) the intervention period as previously reported^12–14^. Briefly, 500 MDA-MB-231 cells were resuspended in a 0.3% v/v agar layer with 80 mg/dL glucose culture medium, stimulated with HS, and then incubated for 21 days. After the incubation period, the cells were stained with 0.01% w/v crystal violet, and only 3D spheroids formed by more than 20 cells were counted with a stereoscope. The effects of HS used in this study are expressed as means of a minimum of three experiments.

### Statistical analyses

In order to evaluate the effect of treatment, test t for paired data was used for comparing PRE and POST-intervention measurements and effects of HS on cell growth; percentage change (var.%) and effect size, expressed as Cohen’s d (ES), were also reported. To elucidate which variables were significantly associated with growth cells, multiple regression with backward stepwise elimination was performed; global goodness of fitting was expressed as R^2^ adjusted. The predictors considered were age (years), BMI (kg/m^2^), PAL (MET-min/week), ${\dot{\text{V}}\text{O}}_{2\max }$ (mL/min/kg), glucose (mg/dL), testosterone (ng/mL), insulin (microU/mL), HOMA-IR index, IGF-1 (ng/mL), IGFBP3 (µg/mL), IGF-1/IGFBP3 (molar ratio), triglycerides (mg/dL), HDL (mg/dL), LDL (mg/dL), total cholesterol, hs-Troponin (ng/L), creatine kinase (UI/L), hs-CRP (mg/L), MeDiet score. In order to reduce predictor numbers, the variance inflation factor values have been calculated. The multicollinearity analysis revealed three variables to be removed: HOMA-IR index, IGFBP3, and total cholesterol. The variance inflation factor analysis has been conducted again revealing no critical values (i.e. < 10) reported in Supplementary Table S1. Additionally, as a precautionary measure, we employed a multiple regression method with forward stepwise elimination, which is the most suitable approach when there is suspicion of overfitting, as compared to other stepwise techniques^31^.

## Results

Participants’ (N = 30) age and time since diagnosis were 53.6 ± 7.6 years and 10.4 ± 2.9 months, respectively. All participants were free from oncological pathology at baseline (i.e., without active disease confirmed by routine mammographic screening) and non-physically active. Participants’ characteristics at the diagnosis are reported in Natalucci et al.^3^. Briefly, the disease before surgery was at stage 0 in 20%, stage I in 50%, and stage II in 30% of participants; 64.3% was in post-menopausal status; the current endocrine therapy was represented by tamoxifen (26.7%) or an aromatase inhibitor (53.3%).

**Figure 1:**
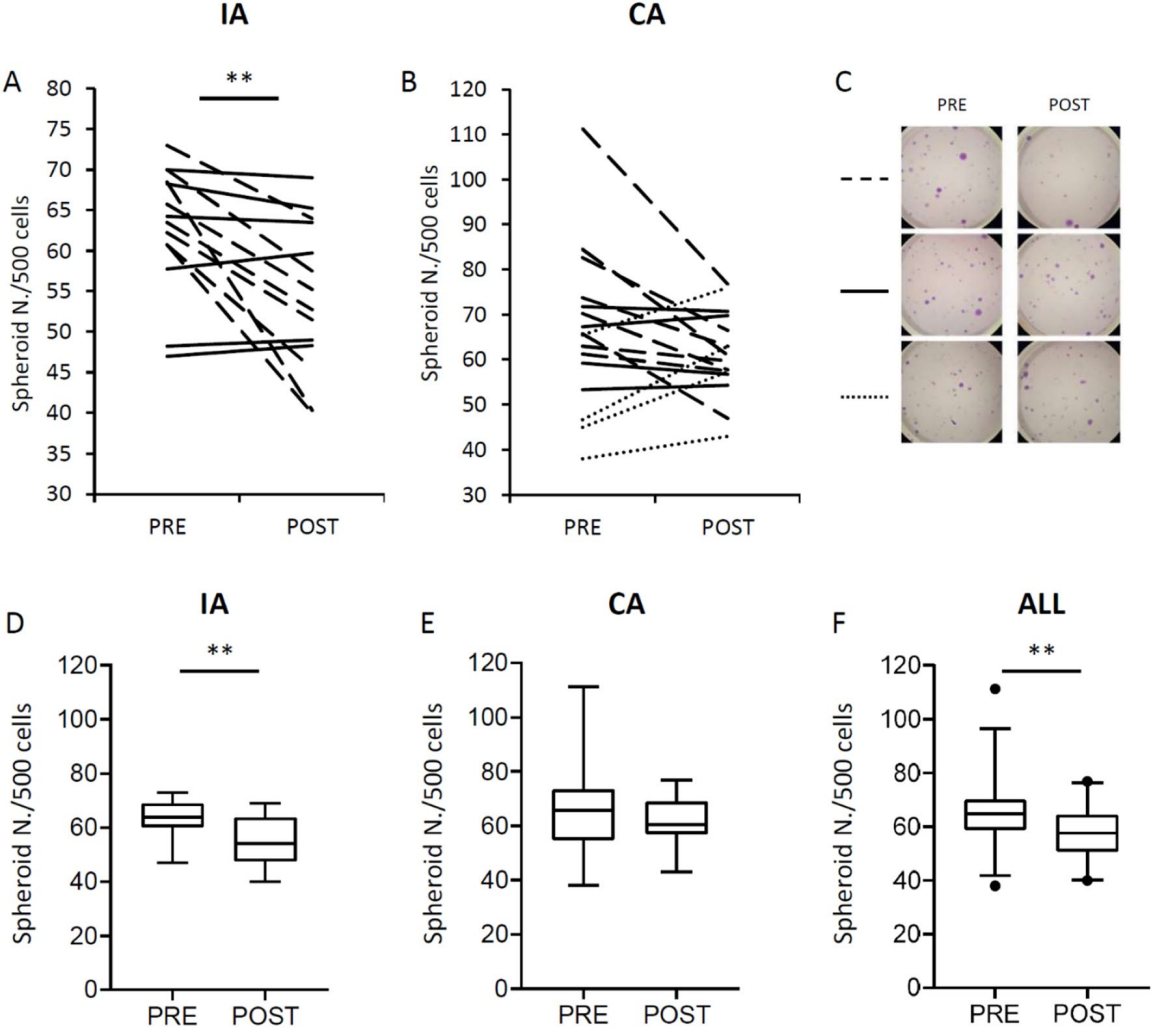
Spheroid formation of TNBC cell line cultured with conditioned sera collected before (PRE) and after (POST) the home-based LI program in the IA (**A,D**), CA (**B,E**), and in all participants (**F**). Spheroids were considered cell colonies formed by more than 20 cells counted after 3-week culture. Dashed lines indicate a reduction of more than 5%; solid lines indicate a variation of less than 5%; dotted lines indicate an increase of more than 5% (**A,B**). Representative photos for dashed, solid, and dotted lines are showed (**C**). Box and Whisker plot of colony numbers; box indicates median and 2nd, and 3rd quartiles (Q2 and Q3); lower whisker indicates Q1–1.5 IR (Interquartile Range); upper whisker indicates Q3 + 1.5 IR; dots indicate outliers. **p < 0.01, paired t-test (**D–F**). *IA* intervention arm, *CA* control arm.

Serum samples collected before and after the LI program were used to evaluate their proliferative potential in the 3D cancer cell model. The ability of TNBC cells to form spheroids (considered as cell colonies composed of more than 20 cells) after stimulation with HS collected before (PRE) and after (POST) the home-based LI was evaluated using MDA-MB-231 cells cultured in semi-solid medium (soft agar) for 21 days. The number of spheroids obtained by incubating cells with HS from each patient is shown in Fig. 1.

The percentage change of spheroid formation capacity induced by HS collected after the LI of IA (POST) in comparison to that collected before (PRE) (var. % POST–PRE) differed more than − 5% in 8 out of 14 participants (− 22.6% on average; Fig. 1A, dashed lines), differed more than + 5% in none out of 14 participants, and did not differ (variations lower than 5%) in 6 out of 14 participants (0.1% on average; Fig. 1A, solid lines). The percentage change of spheroid formation capacity induced by HS collected after the LI of CA (var.% POST–PRE) differed more than − 5% in 8 out of 16 participants (− 18.8% on average; Fig. 1B, dashed lines), differed more than + 5% in 4 out of 16 participants (22.9% on average; Fig. 1B, dotted lines), and did not differ (variations lower than 5%) in 4 out of 16 participants (0.0% on average; Fig. 1B, solid lines). Representative photos from each condition were presented in Fig. 1C. Considering variations observed in all BC survivors, the average percentage change was − 12.8% in the IA (paired t-test between mean colony number; p < 0.01) (Fig. 1D), − 3.7% in the CA (paired t-test between mean colony number; p > 0.01) (Fig. 1E), and − 8.0% overall (paired t-test between mean colony number; p < 0.01) (Fig. 1F).

Physio-metabolic changes were then considered to find correlations with those modifications and cancer cell proliferation. As shown previously, similar adaptations to LI programs in CA and IA were found, showing no significant changes between the two groups in PAL, anthropometric, body composition, and metabolic parameters over time^3^, probably linked to the lack of on-site exercise supervision due to the COVID-19 pandemic confinement.

In contrast, as shown in Table 1, after the 12-week home-based LI intervention, there was an overall significant amelioration in BMI, PAL, and ${\dot{\text{V}}\text{O}}_{2\max }$ values. Moreover, significant decreases in glucose, insulin, HOMA Index, IGFBP3, total cholesterol, LDL, and hs-CRP levels were found (Table 1).

**Table 1:** Comparison between PRE and POST-intervention body composition, physical activity level, and metabolic parameters.

	PRE (Mean ± SD)	POST (Mean ± SD)	Var.%	ES	p-value (t-test)
BMI (kg/m^2^)	26.0 ± 5.0	25.5 ± 4.7	** − 1.92**	**0.16**	**0.035**
PAL (MET-min/week)	647 ± 547	1043 ± 564	**61.21**	**1.13**	** < 0.001**
*V̇*O_2max_ (mL/min/kg)	30.5 ± 5.8	33.4 ± 6.8	**9.51**	**0.71**	** < 0.001**
Glucose (mg/dL)	100.8 ± 11.4	91.7 ± 11.0	** − 9.03**	**1.28**	** < 0.001**
Insulin (microU/mL)	7.92 ± 4.68	6.49 ± 3.94	** − 18.04**	**0.51**	**0.018**
HOMA-IR index	2.07 ± 1.54	1.53 ± 1.11	** − 26.09**	**0.58**	**0.005**
IGF-1 (ng/mL)	164.3 ± 70.9	166.7 ± 57.6	1.46	0.06	0.644
IGFBP3 (µg/mL)	6.06 ± 1.52	4.25 ± 1.40	** − 29.87**	**1.95**	** < 0.001**
IGF-1/IGFBP3 (molar ratio)	99.7 ± 27.1	156.8 ± 50.7	**58.0**	**1.28**	** < 0.001**
Triglycerides (mg/dL)	102.8 ± 43.7	93.3 ± 43.7	** − **9.24	0.34	0.091
Total cholesterol (mg/dL)	217.7 ± 39.3	208.5 ± 37.3	** − 4.23**	**0.38**	**0.029**
HDL (mg/dL)	62.3 ± 15.9	60.8 ± 13.5	** − **2.41	0.16	0.242
LDL (mg/dL)	137.7 ± 29.9	126.1 ± 28.3	** − 8.42**	**0.63**	** < 0.01**
hs-Troponin (ng/L)	3.07 ± 1.14	2.73 ± 2.72	** − **11.07	0.18	0.479
Creatine kinase (UI/L)	109.7 ± 90.7	99.1 ± 43.4	** − **9.66	0.17	0.578
hs-CRP (mg/L)	2.18 ± 2.14	1.75 ± 1.74	** − 19.72**	**0.33**	**0.027**
MeDiet score	6.9 ± 2.3	8.8 ± 2.2	** − 28.02**	**0.95**	** < 0.001**

*ES* effect size, *BMI* body mass index, *PAL* physical activity level, *V*O_2max_ maximal oxygen uptake, *HOMA-IR* homeostasis model assessment of insulin resistance, *IGF-1* insulin-like growth factor 1, *IGFBP3* IGF-1 binding protein 3, *HDL* high-density lipoprotein, *LDL* low-density lipoprotein, *hs-CRP* high-sensitivity C-reactive protein.

Significant values are in bold.

Then, statistical analyses were conducted to find a correlation between assessed physiological and metabolic parameters and spheroid formation capacity induced by sera collected before and after the LI. Since the present work is based on the evaluation of the stimulation of cancer cell growth induced by circulating factors and given the lack of meaningful differences in the modifications on parameters between the two arms^3^, CA and IA were considered as a single group, and statistical analyses were conducted in a PRE-POST setting. Firstly, a regression analysis was performed using only the values collected before the LI intervention; spheroid formation was used as independent variable; predictors are listed in the methods paragraph. The analysis revealed that circulating IGF-1 was the only predictor significantly identified (b, 0.112; p = 0.001) whereas the other parameters were not significantly associated with spheroid formation (Supplementary Table S1). The goodness of fit was 0.526 (R^2^ adjusted), showing that 53% of the explained deviance in colony number is due to IGF-1. Moreover, the IGF-1 beta coefficient of 0.112 implies that the colony number at the initial time (PRE) depends (for the 53% of variance) from IGF-1 value multiplied by 0.112. This unexpected result gave us the rationale to deeply analyze the association between IGF-1 levels and cell proliferation.

**Figure 2:**
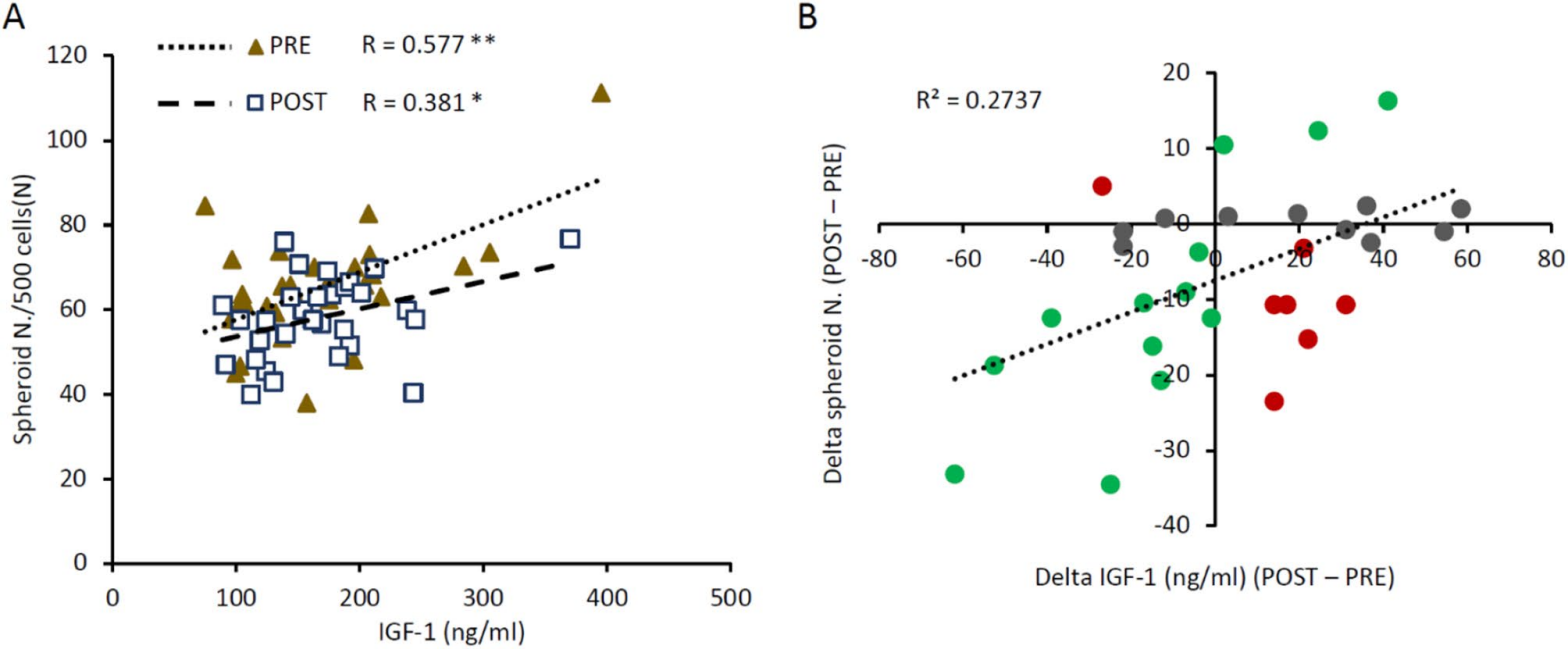
Correlation between IGF-1 and spheroid formation variations. Spheroids were considered cell colonies formed by more than 20 cells after 3-week incubation. (**A**) Correlations between colony number and IGF-1 levels PRE (triangle, p < 0.01) and POST (square, p < 0.05). (**B**) Correlation between delta colony number (POST–PRE) and delta IGF-1 levels (POST–PRE) (p < 0.01). Gray dots: variations in colony number of less than 5%; green dots: positive correlation between colony number and IGF-1 level variations. Red dots: mismatches between variations.

To this aim, we evaluated the variation of IGF-1 levels after the LI period related to the variation of spheroid formation. In Fig. 2A, the correlations between IGF-1 levels and spheroid formation before and after the LI are shown. Before the LI (PRE), a significant correlation between colony formation and IGF-1 levels was revealed (R = 0.577; p < 0.01), confirming results obtained by regression analysis. After the LI (POST), a lower but significant correlation was also found (R = 0.381; p < 0.05).

In Fig. 2B, changes in spheroid formation (Δ POST–PRE) related to changes in IGF-1 levels (Δ POST–PRE) are represented. We observed a positive correlation between the variables (R = 0.523; p < 0.01). Gray dots indicate variations in spheroid formation of less than 5%. Green dots refer to samples where an increase in spheroid formation was related to an increase in IGF-1 levels and samples where a decrease in spheroid formation was related to a decrease in IGF-1 levels.

## Discussion

Consistent epidemiological evidence shows the importance of lifestyle changes for the reduction of BC risk and prevention of BC recurrence. Many published guidelines, such as the American Cancer Society Guideline for Cancer Prevention^21^, the World Cancer Research Fund/American Institute for Cancer Research Report on Cancer^32^, and the ACS/American Society of Clinical Oncology breast cancer survivorship care guideline^33^ recommend regular physical activity and healthy nutrition to prevent cancer development or reduce cancer recurrence. In this context, we have previously evaluated the effects of exercise-conditioned human sera in a 3D cellular model mimicking the biological mechanisms of cancer dormancy in vitro^12–14^. Here, we show the in vitro effects of human sera conditioned by a home-based LI intervention conducted in BC survivors during the first wave of the COVID-19 pandemic.

Serum samples collected before and after the LI intervention were used in this study to evaluate its effects on TNBC cells’ 3D growth in vitro. This experimental model has emerged as a connection between in vitro and in vivo models, representing a reliable method to study the capacity of cells to grow and form 3D colonies in anchorage-independent conditions, which is a characteristic unique to cancer cells, avoiding the use of animals in experimental research^34,35^. Overall, we observed a decrease in spheroid formation induced by serum collected after the intervention program in 53.3%, an increase in 13.3%, and no meaningful differences in 33.3% of participants. Moreover, we found a slight but significant decrease in the average tumorigenic potential of sera collected from participants, especially in the IA, after the LI compared to those collected before, suggesting a possible role of the exercise supervision to have benefit in the proliferative cancer cell control. However, it should be noted that both arms received weekly phone contacts to remind healthy nutritional and exercise lifestyles, and that, due to the COVID-19 confinements, participants in the IA received a weekly phone call to give feedback and receive exercise prescriptions. Thus, the few differences between counselling for CA and the program for the IA could be the reason of a decrease in cell proliferation using sera from participants enrolled in both arms.

Although acute exercise sessions have been extensively shown to have a significant effect on cancer cell proliferation^8,10,12,36,37^, authors showed the efficacy of a training period in reducing cancer cell growth in vitro with uncertain results^38–43^. Particularly, the differences in BMI between the exercise and control groups^38,40,42^ or the combination of regular aerobic exercise and a low-fat diet^39,41,43^ did not allow us to understand if the lower proliferative capacity of prostate cancer cells given by the training-conditioned sera were attributable to the training itself or to other variables included. Similar results were obtained by Schwappacher et al*.*^44^; also, in this case, cancer cells showed a lower proliferative capacity when stimulated by training-conditioned sera. However, blood samples were collected 1-h after the last session, so the effects of training cannot be isolated from the effects of the last bout of exercise. Furthermore, other recent publications highlighted no evidence of an effect of exercise training on cancer cell growth when post-training samples were collected between 1 and 7 days after the last exercise bout^12,36,45^. For these reasons, the results reported in the present study represent the first evidence of the effects of a LI program on modulating the BC cell tumorigenic capacity.

Preclinical studies suggest that systemic changes such as myokine modulation induced by acute exercise can control BC cell viability and proliferation^46^. In contrast, long-term training may reduce systemic risk factor levels such as sex hormones, insulin, and inflammatory markers, but this effect is tightly correlated to weight loss, and there is a lack of causal evidence proving a direct link between exercise training and the risk reductions of cancer and cancer recurrences^46^. The translation approaches could fill this gap by correlating the effects on cell proliferation and metabolic parameters affected by exercise and/or nutritional interventions. The nutritional aspect, indeed, has an important role in the prevention of cancer recurrences. Even though the impact of the Mediterranean diet on BC recurrence and progression is still under investigation^47^, there is evidence that the Mediterranean diet could reduce the recurrence risk of BC by reducing chronic inflammation^48^.

In this study, we conducted a statistical analysis to find predictors of spheroid formation induced by LI-conditioned sera. Interestingly, results suggest that IGF-1 is the only metabolic parameter included in the analysis significantly associated with cellular growth. IGF-1 is a complex system and a well-recognized proliferative inducer^49–51^ that represents a risk factor for BC development and BC recurrences^52^. Moreover, in the observational analyses of UK Biobank data, Murphy et al*.* found that higher circulating concentrations of IGF-I were associated with greater BC risk^53^. Even though the positive association between genetically predicted IGF-1 concentrations and BC risk seems to be restricted to ER+ tumors^53^, other studies reported that, at the biological level, IGF-1/IGF-1R-FAK-YAP network cascade triggers the growth potential of triple-negative BC cells, potentially contributing to the progression of the aggressive TNBC subtype^54,55^. This interesting aspect needs to be deeply analyzed in further studies.

Given its important role in cancer development and progression, the IGF system represents a possible target that can be modulated by physical activity in the pathogenesis of BC^56^. In this context, we recently showed the relationship between circulating IGF-1 and indices of Metabolic syndrome and insulin resistance in BC survivors^57^. Moreover, our results showed that N-linked glycosylation regulates the stability of the IGF-1Ea pro-hormone, controlling the mature IGF-1 production and secretion^51^. As suggested by Runchey et al.^58^ and Brand-Miller et al.^59^, foods with a low glycemic index could have a role in the modulation of IGF-1 axis, reducing its bioavailability and activity. It could be hypothesized that the Mediterranean diet—characterized by low-glycemic index food intake—and glycemia control could modulate the IGF-1 axis and IGF-1-related cancer cell proliferation here reported. Target mechanisms may be represented by a reduction of IGF-1 levels and insulin levels in older cancer survivors, and an increase in IGFBPs levels^60^, the suppression of IGF-1/ER signaling pathway crosstalk, and the modulation of stromal IGF-1 interaction in the tumor microenvironment.

The main limitation of this study is the low number of participants involved. Variations in cell proliferation modulated by lifestyle changes are small, and for a precise quantification, a larger sample is needed. Moreover, as the small sample size makes it difficult to perform a stratified analysis based on the BC stage at the diagnosis, low-risk stage 0 and stages I–III BC were grouped. Even though ductal carcinoma in situ (DCIS), or stage 0 BC, is considered a low-risk cancer, it also has proliferative potential, and up to 40% of DCIS can progress to invasive ductal carcinoma^61^. In this context, although the inclusion of DCIS could represent a potential bias, long-term follow-up studies reported a risk of recurrence up to 30%^62^ and a small risk of mortality in patients with DCIS^63,64^. It is also important to underline that this experimental approach aims to evaluate the effect of a lifestyle intervention in BC survivors without active pathology (i.e., without active disease confirmed by routine mammographic screening at baseline) on circulating molecules that could impact cell proliferation, regardless of the pathology stage assigned at the diagnosis. Evidence here presented could get the basis for setting up an experimental model through which the prediction of the exercise and lifestyle effects could be possible for the prevention of BC recurrences. In fact, cell-based models can offer the advantage of selectively analyzing the role of different molecular pathways by gene editing and knockout. Specifically, further studies can consider the generation of IGF-1 receptor defective cell lines to deeply investigate its role in the proliferative potential of LI and exercise-conditioned serum. In addition, the modification in the IA protocol due to the forced changes by the COVID-19 pandemic did not allow to evaluate the effects of an on-site supervised exercise program. However, the results here reported give important evidence about the role of a LI program, even though home-based, in physio-metabolic changes and cancer cell tumorigenic potential reduction.

In conclusion, the application of translational research in predicting the effect of metabolic and physiological changes induced by exercise and nutritional interventions in BC survivors could be a promising tool for knowledge improvement for tertiary cancer prevention strategies.

### Supplementary Information

Supplementary Figure S1.

Supplementary Table S1.

## Data Availability

The datasets used and/or analyzed during the current study available from the corresponding author on reasonable request.

## References

[1] Moore SC (2016). Association of leisure-time physical activity with risk of 26 types of cancer in 1.44 million adults. JAMA Intern. Med..

[2] Orman A, Johnson DL, Comander A, Brockton N (2020). Breast cancer: A lifestyle medicine approach. Am. J. Lifestyle Med..

[3] Natalucci V (2021). Effects of a home-based lifestyle intervention program on cardiometabolic health in breast cancer survivors during the covid-19 lockdown. J. Clin. Med..

[4] Peterson LL, Ligibel JA (2018). Physical activity and breast cancer: An opportunity to improve outcomes. Curr. Oncol. Rep..

[5] Levin GT, Greenwood KM, Singh F, Newton RU (2018). Modality of exercise influences rate of decrease in depression for cancer survivors with elevated depressive symptomatology. Support. Care Cancer.

[6] Olsson Möller U, Beck I, Rydén L, Malmström M (2019). A comprehensive approach to rehabilitation interventions following breast cancer treatment—A systematic review of systematic reviews. BMC Cancer.

[7] World Cancer Research Fund/American Institute for Cancer Research. *Diet, Nutrition, Physical Activity and Cancer: A Global Perspective. 3rd Export Report. 2018.* https://www.wcrf.org/wp-content/uploads/2021/02/Summary-of-%0AThird-Expert-Report-2018.pdf (2018).

[8] Metcalfe RS (2021). Anti-carcinogenic effects of exercise-conditioned human serum: Evidence, relevance and opportunities. Eur. J. Appl. Physiol..

[9] Brown MJ, Morris MA, Akam EC (2021). An exploration of the role of exercise in modulating breast cancer progression in vitro: A systematic review and meta-analysis. Am. J. Physiol. Cell Physiol..

[10] Orange ST, Jordan AR, Saxton JM (2020). The serological responses to acute exercise in humans reduce cancer cell growth in vitro: A systematic review and meta-analysis. Physiol. Rep..

[11] Yeh AC, Ramaswamy S (2015). Mechanisms of cancer cell dormancy-another hallmark of cancer?. Cancer Res..

[12] Baldelli G (2021). The effects of human sera conditioned by high-intensity exercise sessions and training on the tumorigenic potential of cancer cells. Clin. Transl. Oncol..

[13] De Santi M (2019). A dataset on the effect of exercise-conditioned human sera in three-dimensional breast cancer cell culture. Data Br..

[14] De Santi M (2019). Metformin prevents cell tumorigenesis through autophagy-related cell death. Sci. Rep..

[15] Natalucci V (2023). Movement and health beyond care, MoviS: Study protocol for a randomized clinical trial on nutrition and exercise educational programs for breast cancer survivors. Trials.

[16] Natalucci V (2023). Effect of a lifestyle intervention program’s on breast cancer survivors’ cardiometabolic health: Two-year follow-up. Heliyon.

[17] Craig CL (2003). International physical activity questionnaire: 12-Country reliability and validity. Med. Sci. Sports Exerc..

[18] Lee PH, Macfarlane DJ, Lam TH, Stewart SM (2011). Validity of the international physical activity questionnaire short form (IPAQ-SF): A systematic review. Int. J. Behav. Nutr. Phys. Act..

[19] Villarini A (2012). Lifestyle and breast cancer recurrences: The DIANA-5 trial. Tumori.

[20] Pistelli M (2021). Abstract PD11-03: Assessing the impact of 12 months lifestyle interventions on breast cancer secondary prevention: A modeling approach. Cancer Res..

[21] Rock CL (2022). American Cancer Society nutrition and physical activity guideline for cancer survivors. CA Cancer J. Clin..

[22] Willett WC (1995). Mediterranean diet pyramid: A cultural model for healthy eating. Am. J. Clin. Nutr..

[23] Villarini A, Villarini M, Gargano G, Moretti M, Berrino F (2015). DianaWeb: A demonstration project to improve breast cancer prognosis through lifestyles. Epidemiol. Prev..

[24] Gianfredi V (2020). E-Coaching: The DianaWeb study to prevent breast cancer recurrences. Clin. Ter..

[25] Ferri Marini C (2021). Assessing maximal oxygen uptake: Creating personalized incremental exercise protocols simply and quickly. Strength Cond. J..

[26] Riebe D, Ehrman J, Liguori G, Magal M, American College of Sports Medicine (2018). ACSM’s Guidelines for Exercise Testing and Prescription.

[27] Gellish RL (2007). Longitudinal modeling of the relationship between age and maximal heart rate. Med. Sci. Sports Exerc..

[28] Jones LW, Eves ND, Haykowsky M, Joy AA, Douglas PS (2008). Cardiorespiratory exercise testing in clinical oncology research: Systematic review and practice recommendations. Lancet Oncol..

[29] Friedrich N (2014). Age- and sex-specific reference intervals across life span for insulin-like growth factor binding protein 3 (IGFBP-3) and the IGF-I to IGFBP-3 ratio measured by new automated chemiluminescence assays. J. Clin. Endocrinol. Metab..

[30] Martínez-González MA (2012). A 14-item Mediterranean diet assessment tool and obesity indexes among high-risk subjects: The PREDIMED trial. PLoS ONE.

[31] Cohen J, Cohen P, West SG, Aiken LS (2013). Applied Multiple Regression/Correlation Analysis for the Behavioral Sciences.

[32] Clinton SK, Giovannucci EL, Hursting SD (2020). The World Cancer Research Fund/American Institute for Cancer Research Third Expert Report on diet, nutrition, physical activity, and cancer: Impact and future directions. J. Nutr..

[33] Runowicz CD (2016). American Cancer Society/American Society of Clinical Oncology Breast Cancer survivorship care guideline. CA Cancer J. Clin..

[34] Singh M, Mukundan S, Jaramillo M, Oesterreich S, Sant S (2016). Three-dimensional breast cancer models mimic hallmarks of size-induced tumor progression. Cancer Res..

[35] Urzì O (2023). Three-dimensional cell cultures: The bridge between in vitro and in vivo models. Int. J. Mol. Sci..

[36] Dethlefsen C (2016). Exercise regulates breast cancer cell viability: Systemic training adaptations versus acute exercise responses. Breast Cancer Res. Treat..

[37] Dethlefsen C (2017). Exercise-induced catecholamines activate the hippo tumor suppressor pathway to reduce risks of breast cancer development. Cancer Res..

[38] Barnard RJ, Ngo TH, Leung PS, Aronson WJ, Golding LA (2003). A low-fat diet and/or strenuous exercise alters the IGF axis in vivo and reduces prostate tumor cell growth in vitro. Prostate.

[39] Barnard RJ, Gonzalez JH, Liva ME, Ngo TH (2006). Effects of a low-fat, high-fiber diet and exercise program on breast cancer risk factors in vivo and tumor cell growth and apoptosis in vitro. Nutr. Cancer.

[40] Barnard RJ, Leung PS, Aronson WJ, Cohen P, Golding LA (2007). A mechanism to explain how regular exercise might reduce the risk for clinical prostate cancer. Eur. J. Cancer Prev..

[41] Tymchuk CN, Barnard RJ, Heber D, Aronson WJ (2001). Evidence of an inhibitory effect of diet and exercise on prostate cancer cell growth. J. Urol..

[42] Tymchuk CN, Barnard RJ, Ngo TH, Aronson WJ (2002). Role of testosterone, estradiol, and insulin in diet- and exercise-induced reductions in serum-stimulated prostate cancer cell growth in vitro. Nutr. Cancer.

[43] Ngo TH, Barnard RJ, Leung PS, Cohen P, Aronson WJ (2003). Insulin-like growth factor I (IGF-I) and IGF binding protein-1 modulate prostate cancer cell growth and apoptosis: Possible mediators for the effects of diet and exercise on cancer cell survival. Endocrinology.

[44] Schwappacher R (2020). Physical activity and advanced cancer: Evidence of exercise-sensitive genes regulating prostate cancer cell proliferation and apoptosis. J. Physiol..

[45] Devin JL (2019). Acute high intensity interval exercise reduces colon cancer cell growth. J. Physiol..

[46] Dethlefsen C, Pedersen KS, Hojman P (2017). Every exercise bout matters: Linking systemic exercise responses to breast cancer control. Breast Cancer Res. Treat..

[47] Mantzorou M (2022). Adherence to mediterranean diet and nutritional status in women with breast cancer: What is their impact on disease progression and recurrence-free patients’ survival?. Curr. Oncol..

[48] Pannu MK, Constantinou C (2023). Inflammation, nutrition, and clinical outcomes in breast cancer survivors: A narrative review. Curr. Nutr. Rep..

[49] De Santi M (2016). Human IGF1 pro-forms induce breast cancer cell proliferation via the IGF1 receptor. Cell. Oncol..

[50] Annibalini G (2016). MIR retroposon exonization promotes evolutionary variability and generates species-specific expression of IGF-1 splice variants. Biochim. Biophys. Acta Gene Regul. Mech..

[51] Annibalini G (2018). The intrinsically disordered E-domains regulate the IGF-1 prohormones stability, subcellular localisation and secretion. Sci. Rep..

[52] Key TJ, Appleby PN, Reeves GK, Roddam AW, Endogenous Hormones and Breast Cancer Collaborative Group (2010). Insulin-like growth factor 1 (IGF1), IGF binding protein 3 (IGFBP3), and breast cancer risk: Pooled individual data analysis of 17 prospective studies. Lancet Oncol..

[53] Murphy N (2020). Insulin-like growth factor-1, insulin-like growth factor-binding protein-3, and breast cancer risk: Observational and Mendelian randomization analyses with ∼ 430,000 women. Ann. Oncol..

[54] Rigiracciolo DC (2020). IGF-1/IGF-1R/FAK/YAP transduction signaling prompts growth effects in triple-negative breast cancer (TNBC) cells. Cells.

[55] Xue L (2021). Metformin and an insulin/IGF-1 receptor inhibitor are synergistic in blocking growth of triple-negative breast cancer. Breast Cancer Res. Treat..

[56] Han JK, Kim G (2021). Role of physical exercise in modulating the insulin-like growth factor system for improving breast cancer outcomes: A meta-analysis. Exp. Gerontol..

[57] De Santi M (2023). Association between metabolic syndrome, insulin resistance, and IGF-1 in breast cancer survivors of DIANA-5 study. J. Cancer Res. Clin. Oncol..

[58] Runchey SS (2012). Glycemic load effect on fasting and post-prandial serum glucose, insulin, IGF-1 and IGFBP-3 in a randomized, controlled feeding study. Eur. J. Clin. Nutr..

[59] Brand-Miller JC, Liu V, Petocz P, Baxter RC (2005). The glycemic index of foods influences postprandial insulin-like growth factor-binding protein responses in lean young subjects. Am. J. Clin. Nutr..

[60] de Boer MC, Wörner EA, Verlaan D, van Leeuwen PAM (2017). The mechanisms and effects of physical activity on breast cancer. Clin. Breast Cancer.

[61] McCormick B (2015). RTOG 9804: A prospective randomized trial for good-risk ductal carcinoma in situ comparing radiotherapy with observation. J. Clin. Oncol..

[62] van Bekkum S, Drukker C, van Rosmalen J, Menke-Pluijmers MBE, Westenend PJ (2023). A low risk of recurrence after breast-conserving surgery for DCIS: A single-institution experience. Cancer Treat. Res. Commun..

[63] Narod SA, Iqbal J, Giannakeas V, Sopik V, Sun P (2015). Breast cancer mortality after a diagnosis of ductal carcinoma in situ. JAMA Oncol..

[64] O’Keefe TJ, Chau H, Harismendy O, Wallace AM (2023). Risk factors for breast cancer mortality after ductal carcinoma in situ diagnosis differ from those for invasive recurrence. Surgery.

