# Supplementary material for: A home-based lifestyle intervention program reduces the tumorigenic potential of triple-negative breast cancer cells
Source: Sci Rep. 2024 Jan 29;14:2409. doi: 10.1038/s41598-024-52065-9 (PMC10824728; doi:10.1038/s41598-024-52065-9)
Supplement: Supplementary file 1 — Supplementary Figure S1. [file 41598_2024_52065_MOESM1_ESM.docx]

Excluded (n=5)

- Not meeting inclusion criteria (n=4)
- Declined to participate (n=1)

Allocated to intervention group (n=14)

- Received Lifestyle intervention (phase I):

nutritional and exercise educational counseling

**+**

MoviS training (3-month) (Phase II):

- Aerobic training (3-week):
- 2 days∙week^-1^ OS
- 1 day∙week^-1^ RS
- 20 to 35 min
- 40 to 50% HRR
- Home-based aerobic training (9-week):
- 3 days∙week^-1^ RS
- 40 to 60 min
- 50 to 70% HRR
- Weekly healthy lifestyle reminder

Allocated to control group (n=16)

- Received Lifestyle intervention (phase I):

nutritional and exercise educational counseling

- Weekly healthy lifestyle reminder

Randomization (n=30)

Cardiometabolic parameters assessment and Serum samples (n=30)

Enrolled and assessed for eligibility (n=35)

Cardiometabolic parameters assessment and Serum samples (n=30)

Stimulation of TNBC cells with human sera sampled before (PRE) and after (POST) the Lifestyle interventions.

Analysis of the associations between serum-induced microtumor formation and anthropometric, body composition, PAL, and metabolic parameters.

**Supplementary Figure S1**: Study design flow diagram. RS, remotely supervised; OS, on-site supervised; HRR, heart rate reserve; TNBC, triple-negative breast cancer; PAL, physical activity level.
