# Supplementary material for: A home-based lifestyle intervention program reduces the tumorigenic potential of triple-negative breast cancer cells
Source: Sci Rep. 2024 Jan 29;14:2409. doi: 10.1038/s41598-024-52065-9 (PMC10824728; doi:10.1038/s41598-024-52065-9)
Supplement: Supplementary file 2 — Supplementary Table S1. [file 41598_2024_52065_MOESM2_ESM.docx]

**Supplementary Table S1**. Results of multiple linear regression with forward stepwise elimination using cell proliferation at Initial time (PRE) as the dependent variable.

| Predictors | b * | SE (b) * | t * | p. * | VIF |
| --- | --- | --- | --- | --- | --- |
| MeDiet score | - | - | - | - | 3.105 |
| Age (years) | - | - | - | - | 4.118 |
| BMI (kg/m2) | - | - | - | - | 5.658 |
| PAL (MET-min/week) | - | - | - | - | 2.833 |
| *V̇*O_2max_ (mL∙min-1∙kg-1) | - | - | - | - | 4.504 |
| Glucose (mg/dL) | - | - | - | - | 6.675 |
| Testosterone (ng/ml) | - | - | - | - | 1.825 |
| Insulin (microU/mL) | - | - | - | - | 4.301 |
| IGF-1 (ng/mL) | 0.112 | 0.03 | 3.739 | 0.001 | 4.284 |
| IGF-1/IGFBP3 (molar ratio) | - | - | - | - | 4.313 |
| Triglycerides (mg/dL) | - | - | - | - | 2.344 |
| HDL (mg/dL) | - | - | - | - | 2.747 |
| LDL (mg/dL) | - | - | - | - | 3.162 |
| hs-Troponin (ng/L) | - | - | - | - | 2.635 |
| Creatine kinase (UI/L) | - | - | - | - | 1.456 |
| hs-CRP (mg/L) | - | - | - | - | 2.689 |

Abbreviations: b, unstandardized beta coefficient; SE (b), standard error of b; t, t-value of the regression coefficient t-test; p, probability value associated with t; VIF. variance inflation factor; BMI, Body Mass Index; PAL, physical Activity Level; *V̇*O_2max_, maximal oxygen uptake; IGF-1, Insulin-like Growth Factor 1; IGFBP3, Insulin-like Growth Factor Binding Protein 3; HDL, High-density Lipoprotein; LDL, Low-density lipoprotein, hs-CRP, High-sensitivity C-reactive Protein. Only values with p > 0.05 are reported.
